# Supplementary material for: Modeling glioblastoma heterogeneity as a dynamic network of cell states
Source: Mol Syst Biol. 2021 Sep 16;17(9):e10105. doi: 10.15252/msb.202010105 (PMC8444284; doi:10.15252/msb.202010105)
Supplement: Supplementary file 6 — Source Data for Figure 5 [file MSB-17-e10105-s004.zip › Figure5A_sourcedata/GSEA_3071/nosigdb_stateVI.GseaPreranked.1622795169056/RICHARDS_NOWAKOWSKI_SCIENCE_2017_IN_CTX_CGE2_UPREG.html]

Details for gene set RICHARDS\_NOWAKOWSKI\_SCIENCE\_2017\_IN\_CTX\_CGE2\_UPREG[GSEA]

|  || Dataset | state63071 |
| Phenotype | NoPhenotypeAvailable |
| Upregulated in class | na\_neg |
| GeneSet | RICHARDS\_NOWAKOWSKI\_SCIENCE\_2017\_IN\_CTX\_CGE2\_UPREG |
| Enrichment Score (ES) | -0.54050714 |
| Normalized Enrichment Score (NES) | -2.6835098 |
| Nominal p-value | 0.0 |
| FDR q-value | 0.0 |
| FWER p-Value | 0.0 |
Table: GSEA Results Summary

  

Fig 1: Enrichment plot: RICHARDS\_NOWAKOWSKI\_SCIENCE\_2017\_IN\_CTX\_CGE2\_UPREG      
 Profile of the Running ES Score & Positions of GeneSet Members on the Rank Ordered List

  

| PROBE | GENE SYMBOL | GENE\_TITLE | RANK IN GENE LIST | RANK METRIC SCORE | RUNNING ES | CORE ENRICHMENT || 1 | CALB2 |  |  | 116 | 0.711 | -0.0230 | No |
| 2 | NR2F1 |  |  | 119 | 0.709 | -0.0137 | No |
| 3 | ANK3 |  |  | 165 | 0.638 | -0.0176 | No |
| 4 | RIMS1 |  |  | 194 | 0.606 | -0.0172 | No |
| 5 | DRG1 |  |  | 231 | 0.580 | -0.0193 | No |
| 6 | EIF5A |  |  | 361 | 0.507 | -0.0488 | No |
| 7 | SLIRP |  |  | 384 | 0.495 | -0.0482 | No |
| 8 | NOP58 |  |  | 439 | 0.475 | -0.0569 | No |
| 9 | NR2F2 |  |  | 507 | 0.453 | -0.0696 | No |
| 10 | KITLG |  |  | 584 | 0.430 | -0.0851 | No |
| 11 | EIF3J |  |  | 664 | 0.408 | -0.1019 | No |
| 12 | ARL4C |  |  | 666 | 0.408 | -0.0965 | No |
| 13 | ITGB1BP1 |  |  | 765 | 0.383 | -0.1189 | No |
| 14 | FAM204A |  |  | 852 | 0.368 | -0.1382 | No |
| 15 | EMC4 |  |  | 1017 | 0.342 | -0.1798 | No |
| 16 | HSPA9 |  |  | 1087 | 0.333 | -0.1947 | No |
| 17 | CCT3 |  |  | 1107 | 0.329 | -0.1956 | No |
| 18 | GTF3C6 |  |  | 1162 | 0.321 | -0.2064 | No |
| 19 | ABRACL |  |  | 1354 | 0.297 | -0.2563 | No |
| 20 | VDAC1 |  |  | 1362 | 0.296 | -0.2542 | No |
| 21 | UQCRB |  |  | 1402 | 0.292 | -0.2612 | No |
| 22 | SSRP1 |  |  | 1615 | 0.268 | -0.3175 | No |
| 23 | MCFD2 |  |  | 1697 | 0.260 | -0.3368 | No |
| 24 | SNRPA1 |  |  | 1720 | 0.259 | -0.3395 | No |
| 25 | SF3B1 |  |  | 1831 | -0.254 | -0.3671 | No |
| 26 | PAK3 |  |  | 1870 | -0.260 | -0.3742 | No |
| 27 | UBXN7 |  |  | 1881 | -0.261 | -0.3735 | No |
| 28 | BOD1L1 |  |  | 1893 | -0.263 | -0.3729 | No |
| 29 | GNPTAB |  |  | 1971 | -0.274 | -0.3909 | No |
| 30 | STT3B |  |  | 2026 | -0.282 | -0.4023 | No |
| 31 | TNRC6C |  |  | 2040 | -0.284 | -0.4021 | No |
| 32 | MSL1 |  |  | 2079 | -0.291 | -0.4088 | No |
| 33 | CGGBP1 |  |  | 2095 | -0.294 | -0.4089 | No |
| 34 | NPAS3 |  |  | 2184 | -0.312 | -0.4295 | No |
| 35 | DCUN1D1 |  |  | 2248 | -0.323 | -0.4429 | No |
| 36 | LUC7L |  |  | 2293 | -0.331 | -0.4507 | No |
| 37 | APC |  |  | 2301 | -0.333 | -0.4481 | No |
| 38 | KIAA1841 |  |  | 2315 | -0.336 | -0.4471 | No |
| 39 | TMOD2 |  |  | 2338 | -0.340 | -0.4487 | No |
| 40 | ZNF302 |  |  | 2396 | -0.353 | -0.4599 | No |
| 41 | SETD2 |  |  | 2459 | -0.368 | -0.4724 | No |
| 42 | ATAD5 |  |  | 2574 | -0.392 | -0.4992 | No |
| 43 | ELOVL6 |  |  | 2575 | -0.392 | -0.4938 | No |
| 44 | KIAA0232 |  |  | 2603 | -0.402 | -0.4958 | No |
| 45 | DST |  |  | 2614 | -0.404 | -0.4931 | No |
| 46 | IGF1R |  |  | 2618 | -0.404 | -0.4883 | No |
| 47 | PAK2 |  |  | 2635 | -0.408 | -0.4872 | No |
| 48 | BAG5 |  |  | 2680 | -0.419 | -0.4939 | No |
| 49 | PDZRN3 |  |  | 2685 | -0.421 | -0.4892 | No |
| 50 | RAB11B |  |  | 2701 | -0.426 | -0.4875 | No |
| 51 | RTN1 |  |  | 2785 | -0.451 | -0.5048 | No |
| 52 | KANSL1 |  |  | 2800 | -0.459 | -0.5024 | No |
| 53 | GNG2 |  |  | 2802 | -0.459 | -0.4963 | No |
| 54 | QKI |  |  | 2844 | -0.474 | -0.5014 | No |
| 55 | PRKCA |  |  | 2847 | -0.474 | -0.4954 | No |
| 56 | ARX |  |  | 2960 | -0.520 | -0.5199 | No |
| 57 | PAIP2 |  |  | 3034 | -0.556 | -0.5328 | Yes |
| 58 | ASAH1 |  |  | 3052 | -0.564 | -0.5298 | Yes |
| 59 | SOX4 |  |  | 3066 | -0.571 | -0.5256 | Yes |
| 60 | SMC1A |  |  | 3067 | -0.571 | -0.5177 | Yes |
| 61 | ARID1B |  |  | 3108 | -0.592 | -0.5208 | Yes |
| 62 | PCDH9 |  |  | 3113 | -0.594 | -0.5137 | Yes |
| 63 | NRXN3 |  |  | 3127 | -0.603 | -0.5091 | Yes |
| 64 | WWP1 |  |  | 3153 | -0.621 | -0.5075 | Yes |
| 65 | CADPS |  |  | 3161 | -0.629 | -0.5008 | Yes |
| 66 | PDS5B |  |  | 3177 | -0.641 | -0.4962 | Yes |
| 67 | INTU |  |  | 3186 | -0.650 | -0.4894 | Yes |
| 68 | CRIP2 |  |  | 3230 | -0.697 | -0.4920 | Yes |
| 69 | ASAP1 |  |  | 3247 | -0.711 | -0.4866 | Yes |
| 70 | BRD3 |  |  | 3260 | -0.721 | -0.4801 | Yes |
| 71 | MAGI1 |  |  | 3265 | -0.726 | -0.4711 | Yes |
| 72 | GAD1 |  |  | 3266 | -0.726 | -0.4611 | Yes |
| 73 | DLX6 |  |  | 3289 | -0.749 | -0.4570 | Yes |
| 74 | CXCR4 |  |  | 3302 | -0.759 | -0.4498 | Yes |
| 75 | SOGA1 |  |  | 3319 | -0.788 | -0.4435 | Yes |
| 76 | GAS2L3 |  |  | 3321 | -0.790 | -0.4328 | Yes |
| 77 | PDP1 |  |  | 3362 | -0.826 | -0.4327 | Yes |
| 78 | SLAIN1 |  |  | 3385 | -0.868 | -0.4269 | Yes |
| 79 | ZEB2 |  |  | 3395 | -0.881 | -0.4173 | Yes |
| 80 | GNAQ |  |  | 3406 | -0.909 | -0.4075 | Yes |
| 81 | CCDC88A |  |  | 3421 | -0.944 | -0.3984 | Yes |
| 82 | ZNF704 |  |  | 3448 | -1.004 | -0.3919 | Yes |
| 83 | TMTC4 |  |  | 3450 | -1.006 | -0.3782 | Yes |
| 84 | CHD7 |  |  | 3454 | -1.017 | -0.3650 | Yes |
| 85 | PIAS2 |  |  | 3455 | -1.018 | -0.3509 | Yes |
| 86 | SLC5A3 |  |  | 3465 | -1.041 | -0.3391 | Yes |
| 87 | ROBO1 |  |  | 3473 | -1.052 | -0.3265 | Yes |
| 88 | SDK2 |  |  | 3489 | -1.088 | -0.3157 | Yes |
| 89 | RAB3IP |  |  | 3493 | -1.094 | -0.3014 | Yes |
| 90 | RGS12 |  |  | 3495 | -1.106 | -0.2864 | Yes |
| 91 | RNF180 |  |  | 3497 | -1.113 | -0.2712 | Yes |
| 92 | NFIB |  |  | 3508 | -1.145 | -0.2582 | Yes |
| 93 | NCAM1 |  |  | 3514 | -1.168 | -0.2435 | Yes |
| 94 | DCLK2 |  |  | 3524 | -1.206 | -0.2293 | Yes |
| 95 | TCF4 |  |  | 3528 | -1.214 | -0.2134 | Yes |
| 96 | PROX1 |  |  | 3531 | -1.223 | -0.1970 | Yes |
| 97 | CDH2 |  |  | 3543 | -1.297 | -0.1822 | Yes |
| 98 | SLCO5A1 |  |  | 3546 | -1.322 | -0.1644 | Yes |
| 99 | CHL1 |  |  | 3570 | -1.443 | -0.1510 | Yes |
| 100 | CADM1 |  |  | 3581 | -1.591 | -0.1318 | Yes |
| 101 | DLX1 |  |  | 3593 | -1.696 | -0.1114 | Yes |
| 102 | DLX2 |  |  | 3595 | -1.720 | -0.0879 | Yes |
| 103 | LRRN3 |  |  | 3606 | -1.871 | -0.0648 | Yes |
| 104 | DLX5 |  |  | 3623 | -2.501 | -0.0348 | Yes |
| 105 | DCX |  |  | 3630 | -2.797 | 0.0023 | Yes |
Table: GSEA details [plain text format]

  

Fig 2: RICHARDS\_NOWAKOWSKI\_SCIENCE\_2017\_IN\_CTX\_CGE2\_UPREG: Random ES distribution      
 Gene set null distribution of ES for **RICHARDS\_NOWAKOWSKI\_SCIENCE\_2017\_IN\_CTX\_CGE2\_UPREG**
